# Application of Convolutional Neural Network-Based Detection Methods in Fresh Fruit Production: A Comprehensive Review

**DOI:** 10.3389/fpls.2022.868745

**Published:** 2022-05-16

**Authors:** Chenglin Wang, Suchun Liu, Yawei Wang, Juntao Xiong, Zhaoguo Zhang, Bo Zhao, Lufeng Luo, Guichao Lin, Peng He

**Affiliations:** ^1^Faculty of Modern Agricultural Engineering, Kunming University of Science and Technology, Kunming, China; ^2^School of Intelligent Manufacturing Engineering, Chongqing University of Arts and Sciences, Chongqing, China; ^3^College of Mathematics and Informatics, South China Agricultural University, Guangzhou, China; ^4^Chinese Academy of Agricultural Mechanization Sciences, Beijing, China; ^5^School of Mechatronic Engineering and Automation, Foshan University, Foshan, China; ^6^School of Mechanical and Electrical Engineering, Zhongkai University of Agriculture and Engineering, Guangzhou, China; ^7^School of Electronic and Information Engineering, Taizhou University, Taizhou, China

**Keywords:** computer vision, deep learning, convolutional neural network, fruit detection, fruit production

## Abstract

As one of the representative algorithms of deep learning, a convolutional neural network (CNN) with the advantage of local perception and parameter sharing has been rapidly developed. CNN-based detection technology has been widely used in computer vision, natural language processing, and other fields. Fresh fruit production is an important socioeconomic activity, where CNN-based deep learning detection technology has been successfully applied to its important links. To the best of our knowledge, this review is the first on the whole production process of fresh fruit. We first introduced the network architecture and implementation principle of CNN and described the training process of a CNN-based deep learning model in detail. A large number of articles were investigated, which have made breakthroughs in response to challenges using CNN-based deep learning detection technology in important links of fresh fruit production including fruit flower detection, fruit detection, fruit harvesting, and fruit grading. Object detection based on CNN deep learning was elaborated from data acquisition to model training, and different detection methods based on CNN deep learning were compared in each link of the fresh fruit production. The investigation results of this review show that improved CNN deep learning models can give full play to detection potential by combining with the characteristics of each link of fruit production. The investigation results also imply that CNN-based detection may penetrate the challenges created by environmental issues, new area exploration, and multiple task execution of fresh fruit production in the future.

## Introduction

Fresh fruits in the market are beloved by people because of their enticing aroma and unique flavor. From fruit flowers blooming to fruit grading, every link of fresh fruit production needs to be seriously supervised so that fruits enter the market without economic loss. In recent years, the world agricultural population and labor force have been having a declining trend leading to the urgent need for automation of fresh fruit production (Yuan et al., 2017). Object detection based on computer vision has been applied to the main link of automatic fresh fruit production such as smart yield prediction, automatic harvesting robots, and intelligent fruit quality grading (Naranjo-Torres et al., 2020).

A function of ML is to ensure that machines can automatically detect objects accurately. Although ML has been applied in many fields, the ML technology has been developing to achieve efficient detection. The detection performance of traditional ML will not improve with increase in training sample data. The features need to be given artificially for object detection, which is also a disadvantage of traditional ML (Mohsen et al., 2021). As an intelligent algorithm in the development of ML, DL has significant advantages over traditional algorithms of ML. The detection performance of DL usually improves with increase in the amount of training sample data. DL can automatically extract features of a detected object using network structure. However, DL takes a lot of training time and runs on computers with higher cost configurations compared with traditional ML (Joe et al., 2022).

Deep learning is a further study on artificial neural networks such as deep belief network (Hinton et al., 2006), recurrent neural network (Schuster and Paliwal, 1997), and convolutional neural network (LeCun et al., 1989). The deep learning algorithm has a similar calculation principle with a mechanism of the visual cortex of animals (Rehman et al., 2019). The deep learning-based technology has broad applications in many domains due to its superior performance in operation speed and accuracy, for example, in the medical field (Gupta et al., 2019; Zhao Q. et al., 2019), in the aerospace field (Dong Y. et al., 2021), in the transportation sector (Nguyen et al., 2018), in the agriculture field (Kamilaris and Prenafeta-Boldú, 2018), and in the biochemistry field (Angermueller et al., 2016).

A CNN with a convolutional layer and a pooling layer was proposed by Fukushima (1980), which was subsequently improved to LeNet (LeCun et al., 1998), GoogleNet (Szegedy et al., 2015), ResNet (He et al., 2016), AlexNet (Krizhevsky et al., 2017), and so on. With the appearance of R-CNN (Girshick et al., 2014), CNN-based object detection became a hot research topic on computer vision and digital image processing (Zhao Z. et al., 2019). Object detection is the coalition of object classification and object location requiring a network to differentiate an object region from the background and accomplish the classification and location of the object. The technique of CNN-based image segmentation using a CNN model to perceive the representative object of each pixel for classifying and locating objects can be performed for object detection tasks. Frequently used image segmentation models are Mask-R-CNN, U-Net (Ronneberger et al., 2015), SegNet (Badrinarayanan et al., 2017), DeepLab (Chen et al., 2018), and so on.

Early fruit image segmentation algorithms use traditional ML algorithms to identify fruit objects by combining shallow characteristics of fruits such as color, texture, and shape, and mainly included threshold segmentation (Pal and Pal, 1993), DTI (Quinlan, 1986), SVM (Cortes and Vapnik, 1995), cluster analysis (Tsai and Chiu, 2008), and so on. Color traits of fruits are frequently used in fruit detection (Thendral et al., 2014; Zhao et al., 2016). Shape, as an outstanding mark of fruits, is applied to fruit segmentation and recognition (Nyarko et al., 2018; Tan et al., 2018). In addition, spectral features and depth information are applied in fruit detection (Bulanon et al., 2009; Okamoto and Lee, 2009; Gené-Mola et al., 2019a; Lin et al., 2019; Tsoulias et al., 2020). The above methods can detect fruit objects; however, they have certain limitations of features expression for fruit object detection in a complex environment. CNN-based detection technology has been proved to have a potential in fresh fruit production by many studies (Koirala et al., 2019b). Models combined with CNN, for example, CNN + SVM (Dias et al., 2018), CNN + ms-MLP (Bargoti and Underwood, 2017), fuzzing mask R-CNN (Huang et al., 2020), faster R-CNN (Gao et al., 2020), the Alex-FCN model (Wang et al., 2018), and 3D-CNN (Wang et al., 2020), have obtained satisfactory detection results in fruit flower detection, fruit recognition, fruit maturity prediction, and surface defect detection-based fruit grading. These successful studies imply that CNN-based methods can break the technical bottleneck in detection and accelerate the mechanization of fresh fruit production.

**FIGURE 1:**
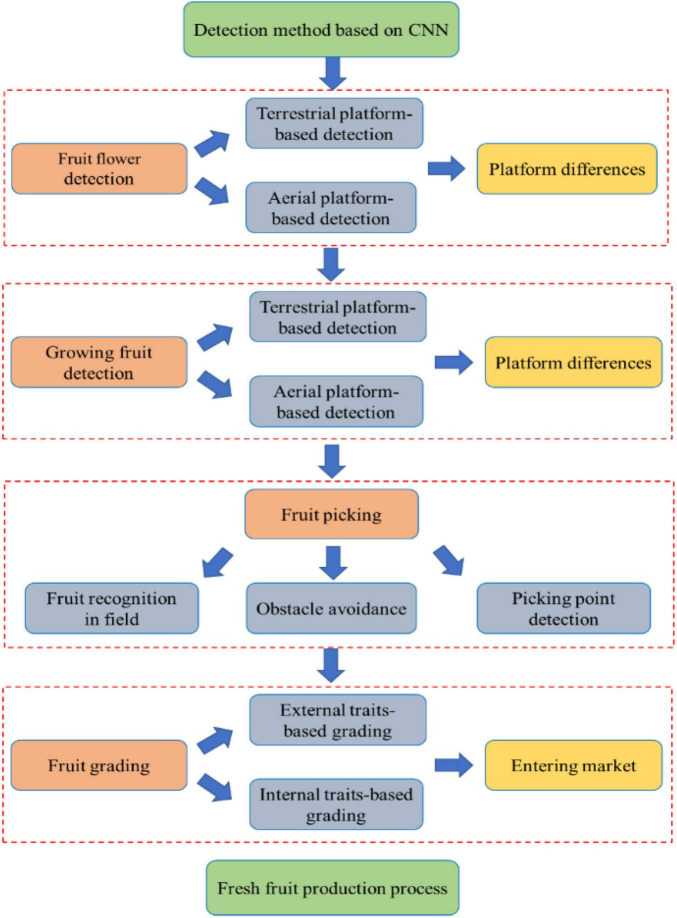
Convolutional neural network (CNN)-based detection application in main links of fresh fruit production.

As shown in Figure 1, this review investigates the CNN-based detection application in the process of fresh fruit production, which is a complete process from fruit flower detection, growing fruit detection, fruit picking to fruit grading. We provide a comprehensive introduction and analysis of the CNN model and its improved models in fresh fruit production. In addition, different CNN-based detection methods are compared and summarized in each link of fresh fruit production. The arrangement of this article is as follows: Section “Common Models and Algorithms of Convolutional Neural Network” introduces the composition and algorithms of CNN; Section “Implementation Process of Convolutional Neural Network-Based Detection” explains the CNN-based detection implementation process; Section “Convolutional Neural Network-Based Fresh Fruit Detection” investigates the current research on CNN applications in each link of fresh fruit production; Section “Challenges and Future Perspective” discusses difficulties that will be encountered by CNN-based detection in future research on fresh fruit production; Section “Conclusion” presents an entire summary of this investigation.

## Common Models and Algorithms of Convolutional Neural Network

### Convolutional Neural Network Models for Image Detection

Common CNN models used for image detection are usually composed of convolutional layers, activation functions, pooling layers, and full-connected layers (Mohsen et al., 2021). A CNN model transforms an image into high dimension information, so a computer can read and extract features from the image. In two-dimensional (2D) convolution operation, each pixel value of an input image entering into a convolutional layer is convoluted with a kernel to generate a feature map. When an input image is three-dimensional (3D) or four-dimensional (4D), a multi-dimension convolution operation will be implemented. In the multi-dimension convolution operation, the channel number of kernels is equal to the channel number of input images, and the channel number of output feature maps is the number of kernels (Alzubaidi et al., 2021). However, in convolutional layers and full-connected layers, the linear connection between the input and the output restricts the ability of a CNN model to solve more complex problems. The activation function is added after the operations of convolution layers and full-connected layers, which can capacitate a CNN model to solve non-linear problems. Common activation functions include the Sigmoid function, the Tanh function, the ReLU function, SoftMax, and so on.

LeNet is the first improved CNN; however, it has not been widely promoted and applied because of simple network structure (LeCun and Bengio, 1995). AlexNet is the first deep CNN architecture and the first CNN model trained on GPU (Krizhevsky et al., 2017). A VGG model with four network structures and different configurations was proposed by the Visual Geometry Group of Oxford University in 2014 (Simonyan and Zisserman, 2014). The most popular network among VGG models is VGG-16 containing thirteen convolutional layers and three full-connected layers. GoogLeNet was a new deep learning structure proposed in 2014 (Szegedy et al., 2015). The most unique of GoogLeNet is the inception component, which utilizes partial connection to accomplish parameter reduction and computation simplicity. A series of inception components including InceptionV2, InceptionV3, and InceptionV4, was proposed for optimizing GoogLeNet (Szegedy et al., 2016). By proving the existence of degradation of CNN while its depth is increasing, ResNet was proposed to improve the CNN by designing residual components with the shortcut connection (He et al., 2016). DenseNet was proposed in 2017, and dense block was the highlight of DenseNet by building connections of all layers with each other to ensure maximum information flow among the layers (Huang et al., 2017). With the popularization of CNN models, it is required that CNN-based image recognition tasks are implemented on mobile terminals or embedded devices. As a lightweight model, MobileNet was designed to run on the CPU platform, and it had good detection accuracy (Howard et al., 2017). These models are fundamentals of CNN-based object detection and can help computers learn more information about images because of functions of feature recognition and extraction. The structure and image detection performance of the above common CNN models are summarized in Table 1.

**TABLE 1:** Structure and performance of common convolutional neural network (CNN) models for image detection.

CNN models	Weight layers	Convolution layer	Kernel size	Active function	Dropout^a^	LRN^b^	BN^c^	Top-5 error (on ImageNet)
AlexNet	8	5	3×3, 5×5, 11×11	ReLU	√	√	–	16.4%
VGG	19	16	3×3	ReLU	√	–	–	7.3%
GoogleNet (Inception-V1)	22	21	1×1, 3×3, 5×5, 7×7	ReLU	√	√	–	6.7%
ResNet	152	151	1×1, 3×3, 7×7	ReLU	√	–	√	3.57%
DenseNet	265	264	1×1, 3×3, 7×7	ReLU	√	–	√	5.29%
MobileNet	28	27	1×1, 3×3	ReLU	–	–	√	*

*^a^Dropout is a training trick, which means that neural network units are temporarily discarded from the network according to a certain probability in the training process of a deep learning network.*

*^b^LRN, local response normalization, is a training trick that can enhance the generalization ability of a model. It creates a competitive mechanism for activities of local neurons, which can make the value of neurons with large responses larger and inhibit neurons with small feedback.*

*^c^BN, batch normalization, normalizes the data of each layer and performs linear transformation to improve data distribution.*

*Means that we have not found relevant data about Mobilenet in the public references.

### Convolutional Neural Network Models for Three-Dimensional Point Cloud Detection

With the development of vision technology, sensors that directly acquire 3D data are becoming more common in robotics, autonomous driving, and virtual/augmented reality applications. Because depth information can eliminate a lot of segmentation ambiguities in 2D images and provides important geometric information, the ability to directly process 3D data is invaluable in these applications. However, 3D data often come in the form of point clouds. Point clouds are typically represented by a set of 3D points that are not arranged in order, each with or without additional features (such as RGB color information). Because of the disordered nature of point clouds and the fact that they are arranged differently from regular mesh-like pixels in 2D images, traditional CNNs struggle to handle this disordered input.

At present, the deep learning point cloud target recognition method mainly has three kinds of point cloud target recognition methods based on views (Kalogerakis et al., 2017), voxels (Riegler et al., 2016), and point clouds (Qi et al., 2017a). Among them, the idea based on views is still to convert three-dimensional data into a two-dimensional representation; that is, 3D data are projected according to different coordinates and different perspectives to obtain a two-dimensional view, and then the two-dimensional image convolution processing method is used to extract features from each view and, finally, aggregate the features to obtain classification and segmentation results. The idea based on voxels is to put an unordered point cloud into the voxel grid, so that it becomes a three-dimensional grid regular data structure, and then as network input data. However, in order to solve problems of view-based and voxel-based computational complexity and information loss, researchers began to consider directly inputting raw point cloud data into the network for processing.

At Stanford University in the United States, Qi et al. (2017a) proposed a new type of neural network, PointNet, for point cloud identification and segmentation directly using a point cloud as the input object, the spatial transformation network T-Net to ensure the displacement invariance of the input point, a shared multilayer perceptron (MLP) to learn the characteristics of each point, and, finally, the maximum pooling layer to aggregate global features. However, PointNet cannot learn the relationship characteristics between different points in the local neighborhood, and then Qi et al. (2017b) proposed PointNet++ to improve PointNet, according to the idea of two-dimensional convolution proposed hierarchical point cloud feature learning for local areas, which is composed of sampling layer, grouping layer and feature extraction layer (PointNet) in the hierarchical module, while improving the stability of the network architecture and the ability to obtain details. Later, the description ability of local features was enhanced in order to make the local structure information between points, such as distance and direction, be able to learn in the network.

**FIGURE 2:**
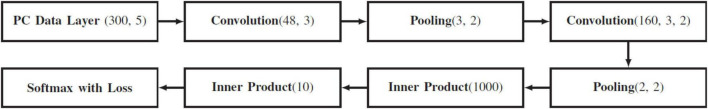
Structure of PointNet.

PointNet inputs an irregular point cloud directly into the deep convolutional network, the framework represents the point cloud as a set of 3D points { (*P*—*i* = l, …, *n*}, where each point *P* is its 3D coordinates plus additional feature channels such as color, normal vector, and other information; the architecture is shown in Figure 2. In response to the point cloud disorder problem, PointNet pointed out that a symmetric method is used; that is, maximum pooling, no matter how many orders there are in *N* points, the maximum eigenvalue in the pooling window corresponding to *N* points is selected for each dimension of the final high-latitude feature and fused into the global feature. For the rotation invariance problem of point cloud, PointNet points out that spacial transform network (STN) is used to solve it. Through the T-Net network to learn the point cloud itself attitude information to obtain a DD rotation matrix (D represents the characteristic dimension), PointNet in the input space transformation using 3×3, feature space transformation using 64×64 to achieve the most effective transformation for the target.

### Convolutional Neural Network-Based Detection Algorithms

Convolutional neural network-based detection algorithms mainly include object detection algorithms, semantic segmentation algorithms, and instance segmentation algorithms, which are described in detail as follows.

#### Object Detection Algorithms

**FIGURE 3:**
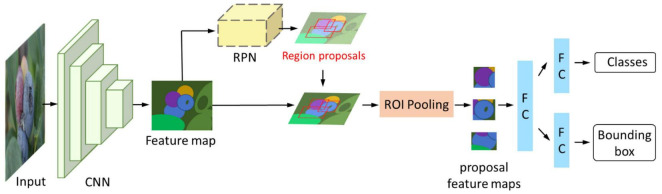
Faster-R-CNN structure. The feature map is extracted by a convolutional neural network, and then the RPN (region proposal network) generates several accurate region proposals according to the feature map. The region proposals are mapped to the feature map. The ROI (region of interest) pooling layer is responsible for collecting proposal boxes and calculating proposal feature maps. Finally, the category of each proposal is predicted through the FC (full connect) layer.

As a kind of object detection algorithm, a two-stage detector is mainly composed of a region proposal generator and classes and bounding box prediction. The R-CNN series is the most representative two-stage detector and includes R-CNN (Girshick et al., 2014), Fast-R-CNN (Girshick, 2015), Faster-R-CNN (Ren et al., 2017), etc. R-CNN is the pioneer in using deep learning for object detection. After that, researchers proposed Fast-R-CNN and Faster-R-CNN in succession to update detection performance. Figure 3 shows the structure of Faster-R-CNN, which is frequently used. Besides the above object detection algorithms, R-FCN and Libra R-CNN are also two-stage detectors.

Compared with a two-stage detector, a one-stage detector conducts classification and bounding box regression after feature extraction without generation of proposal regions. Prediction of objects depends on doing dense sampling on an input picture. Representative one-stage detectors are the YOLO series and SSD (single shot multibox detector). The YOLO series contains YOLOv1 (Redmon et al., 2016), YOLOv2 (Redmon and Farhadi, 2017), YOLOv3 (Redmon and Farhadi, 2018), and YOLOv4 (Bochkovskiy et al., 2020). Notably, during the evolution of YOLO, a new convolution neural net, DarkNet, was constructed for feature extraction. Furthermore, YOLOv2 referenced the anchor conception from Faster-R-CNN. YOLOv3 contains three different output nets that can predict multi-scale pictures. SSD (Liu W. et al., 2016) is also a kind of one-stage detector that can implement multi-box prediction. VGG-16 was used as a backbone in SSD. With the development of DL, more improved one-stage detection algorithms have been designed.

A comparison of CNN models between two-stage detectors and one-stage detectors is shown in Table 2. As can be seen in Table 2, frames per second (FPS) of the one-stage detector are bigger than those of the two-stage detector, which implies that the detection speed of the one-stage detector is faster than that of the two-stage detector. The FPS and mAP of the Mask-R-CNN model are bigger than those of other models of the two-stage detector. It shows that the Mask-R-CNN model has faster detection speed and higher detection accuracy than the two-stage detector. However, in the one-stage detector, no CNN model has faster detection speed and higher detection accuracy. Because of lack of mAP in some CNN models on data of VOC2012 and COCO, the accuracy of the two detectors cannot be compared.

**TABLE 2:** Summary of common CNN-based object detection models.

Type	Name	Backbone	Bounding boxes generation	Additional blocks	FPS^a^	mAP/%		References
						VOC2012^b^	COCO^c^	
Two-stage	R-CNN	AlexNet	SS^d^	–	0.03	59.2	–	Girshick et al., 2014
	Fast-R-CNN	VGG-16	SS+ROI pooling	–	7.00	68.4	19.7	Girshick, 2015
	Faster-R-CNN	VGG-16/ResNet-101	RPN+ROI pooling	–	7.00/5.00	70.4/73.8	21.9/34.9	Ren et al., 2017
	Mask-R-CNN	ResNeXt-101-FPN	RPN+ROI align	FCN	11.00	73.9	39.8	He K. et al., 2017
One-stage	SSD	VGG-16	Anchor	–	19.3	78.5	28.8	Liu W. et al., 2016
	YOLOv1	GoogleNet	–	–	45.0	57.9	–	Redmon et al., 2016
	YOLOv2	DarkNet-19	Anchor	–	40.0	73.5	21.6	Redmon and Farhadi, 2017
	YOLOv3	DarkNet-53	Anchor	FPN, SPP	51.0	–	33.0	Redmon and Farhadi, 2018
	YOLOv4	CSPDarkNet53	Anchor	FPN+PA, SPP	23.0	–	43.5	Bochkovskiy et al., 2020

*^a^FPS, frames per second, is used to measure how many frames (pictures) the target network can detect per second.*

*^b^VOC2012: a dataset used in pattern analysis, statistical modeling, and computational learning visual object classes challenge 2012.*

*^c^COCO: Microsoft Common Objects in Context, a dataset funded and labeled by Microsoft in 2014.*

*^d^SS: selective search (Uijlings et al., 2013).*

#### Semantic Segmentation Algorithms

Unlike box recognition in object detection, semantic segmentation refers to pixel-level recognition and classification, which classifies pixels of the same class into one group. Early DL-based semantics segmentation methods performed clustering to generate super-pixels and a classifier to classify them (Couprie et al., 2013; Farabet et al., 2013). However, such methods have drawbacks of time-consuming and rough segmentation results. With the popularity and development of object detection algorithms based on CNNs, semantic segmentation algorithms have also made great progress, and can be divided into region-classification-based image semantic segmentation and pixel-classification-based image semantic segmentation.

The method of region-classification-based image semantic segmentation first selects the appropriate region, then classifies the pixels in the candidate region. SDS (simultaneous detection and segmentation) is a model based on R-CNN that can simultaneously detect and semantically segment targets (Hariharan et al., 2014). In 2016, based on the SDS method, Liu S. et al. (2016) convoluted images using sliding windows of different sizes and constructed multi-scale feature maps, proposed an MPA (multi-scale patch aggregation) method that can semantically segment an image at the instance level. DeepMask is a segmentation model proposed based on CNN to generate object proposals (Pinheiro et al., 2015). It generates image patches directly from original image data and then generates a segmentation mask for given image patches. The whole process is applied to a complete image to improve the efficiency of segmentation.

The method of pixel-classification-based semantic segmentation does not need to generate object candidate regions but extracts image features and information from labeled images. Based on that information, a segmentation model can learn and infer the classes of pixels in an original image, and classify each pixel in the image directly to achieve end-to-end semantic segmentation. FCN (fully convolutional network) is a popular semantic segmentation model that can be compatible with any size of images (Shelhamer et al., 2017). FCN can distinguish the categories of pixels directly, which greatly promotes the development of semantic segmentation. Subsequently, researchers proposed a series of methods based on FCN. FCN-based image semantic segmentation methods are as follows: DeepLab, DeepLab-V2, and DeepLab-V3. Image semantics segmentation methods based on encoder-decoder model are as follows: U-net, Segnet, Deconvnet, and GCN (global convolution network).

#### Instance Segmentation Algorithms

The purpose of instance segmentation is to distinguish different kinds of objects in an image and different instances of the same kind. Therefore, it has the characteristics of object detection and semantic segmentation at the same time. Because of the characteristics of instance segmentation, it can include instance segmentation based on object detection and instance segmentation based on semantics segmentation.

An instance segmentation algorithm based on object detection has been the mainstream direction in the field of instance segmentation research in recent years. Its main process is to locate an instance using an object detection algorithm, and then segment the instance in each detected box. Mask-R-CNN is one of the famous models in instance segmentation proposed by He K. et al. (2017). Mask-R-CNN is one of the famous models in instance segmentation on the basis of Fast-R-CNN(He K. et al., 2017). As a representative instance segmentation model, many scholars are deeply inspired by Mask-R-CNN. Based on Mask-R-CNN, PANet (path aggregation network) introduces a bottom-up path augmentation structure, adaptive feature pooling, and a fully connected fusion structure to obtain more accurate segmentation results (Liu S. et al., 2018). Chen et al. (2018) proposed Masklab, which uses directional features to segment instances of the same semantic class. In 2019, the first instance segmentation algorithm based on a one-stage object detection algorithm, YOLACT, was proposed by Bolya et al. (2019). It added a mask generation branch behind the one-stage object detector to complete a segmentation task. The overall structure of YOLACT is relatively lightweight, and the trade-off between speed and effect would be good. In addition, there are some newly proposed instance segmentation algorithms such as MS-R-CNN (Huang et al., 2019), BMask-R-CNN (Cheng et al., 2020) and BPR (Tang et al., 2021).

An instance segmentation algorithm based on semantic segmentation classifies each pixel first and then segments different instances of the same category. For example, the SGN (Liu et al., 2017) model decomposes an instance segmentation into multiple subtasks, then uses a series of neural networks to complete these subtasks, and finally recombines the results of the subtasks to obtain the segmentation task.

#### Differences of Detection Algorithms

**FIGURE 4:**
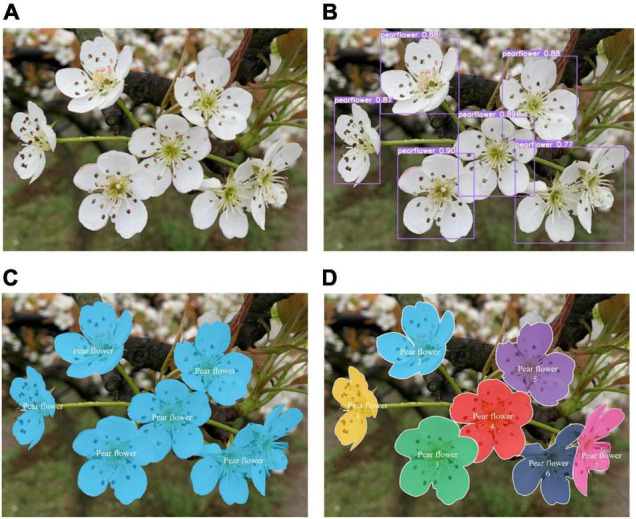
Different CNN-based algorithms for pear flower detection. **(A)** Original image, **(B)** object detection, **(C)** semantic segmentation, and **(D)** instance segmentation.

In this section, differences among object detection, semantic segmentation, and instance segmentation are visually explained through pear flower detection. Figure 4A is an undetected image of pear flowers. The result of detecting pear flowers with the object detection algorithm is shown in Figure 4B, and it shows the approximate position of pear flowers with bounding boxes. The result with semantic segmentation algorithm is shown in Figure 4C, which reaches the pixel level compared with the result of object detection. It means that when labeling data sets, the annotation of the task of semantic segmentation is also at pixel level. Compared with rectangular box annotation in the object detection task, the annotation of semantic segmentation task is more complex. The result with the instance segmentation algorithm is shown in Figure 4D, and the detection results of instance segmentation are more detailed than those of semantic segmentation in distinguishing each pear flower individual.

## Implementation Process of Convolutional Neural Network-Based Detection

This section introduces the main procedures of comprehensively training a CNN-based deep learning model for basic tasks. The first step is determining the learning target and establishing the data set. Second, it is vital to choose an adept deep learning framework to modify the model and implement training. Finally, mastering the estimation metrics of deep learning models leads to knowing the performance of the modified models and training results.

### Data Set Construction

#### Dataset Acquisition

An RGB camera, which can capture the properties of a fruit surface, such as color, shape, defect, and texture, is a pervasive and affordable camera for image acquisition used in many types of research (Fu et al., 2020a). Vasconez et al. (2020) held an RGB camera and acquired apple, avocado, and lemon pictures at 30 frames per second in orchards. However, the information obtained from RGB images is not sufficient for 3D location and reconstruction. Thus, most researchers have begun utilizing RGB-D to capture RGB images and depth images in their experiments. RGB-D cameras generally operate with three depth measurement principles: structured light, time of flight, and active infrared stereo technique (Fu et al., 2020a). Data sets that provide geometric information and radiation information can enhance the models’ ability to distinguish fruits from complex environments. Gené-Mola et al. (2019b) established an apple data set containing multimodal RGB-D images and pointed out that the model provided with RGB-D images is more robust than that provided with RGB images in a complex environment. However, sensors in most depth cameras cannot obtain information beyond 3.5 m, and light detection and ranging (LiDAR) scanners are needed to acquire information at a far distance (Tsoulias et al., 2020). A LiDAR scanner can directly provide three-dimensional positioning information of fruits without being affected by light conditions. In addition, LiDAR data can improve the positioning accuracy of fruits because of the appearance of different objects showing different reflectivity to laser. Gené-Mola et al. (2019a), by detecting Fuji apples in orchards with LiDAR, found that the reflection of apple surface was 0.8 higher than that of leaves and branches at a wavelength of 905 nm.

The internal properties of fruits need hyperspectral reflectance images to be represented. Yu et al. (2018) used a hyperspectral imaging system that constituted of a spectrometer, a CDD camera, a light system, and a computer to detect the internal features of Korla fragrant pear. Some scholars bought a designed hyperspectral system for data collection (Wang et al., 2020).

#### Data Set Augmentation

**FIGURE 5:**
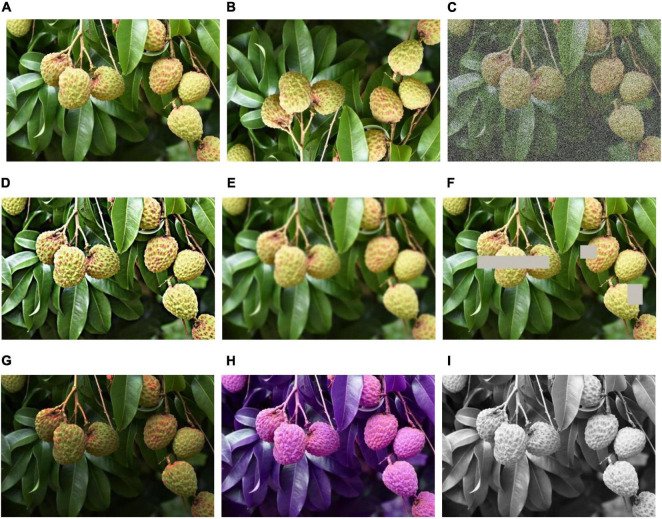
Example images with different image processes. **(A)** Original image, **(B)** vertical flip image, **(C)** noise injected image, **(D)** sharpened image, **(E)** Gaussian blurry image, **(F)** random erased image, **(G)** image with brightness adjustment, **(H)** RGB2GRB image, and **(I)** gray image.

Data sets, as an input, play a significant part in a DL model. Most researchers consider that enhancing the scale and quality of data sets can strengthen the models’ generalization and learning capacity. The methods of dataset augmentation can be divided into the basic-image-manipulation-based method and the DL-based method. The most straightforward and frequently-used methods based on basic image processing are geometric transformations, flipping, color space, cropping, rotation, translation, noise injection, color space transformations, kernel filters, mix images, and random erasing. Figure 5 displays example images with some usual image processes. In addition, the DL-based method contains SMOTE (Chawla et al., 2002), adversarial training, DC-GAN (deep convolutional GAN) (Zheng et al., 2017), CycleGAN (Zhu et al., 2017), CVAE-GAN (Bao et al., 2017), etc.

**FIGURE 6:**
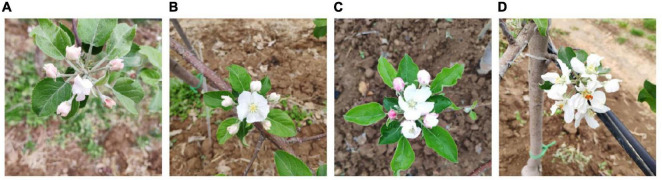
Apple inflorescence: **(A)** the central flower and the side flowers have a bud shape, **(B)** the central flower has a semi-open shape and the side flowers have a bud shape, **(C)** the central flower has a fully open shape and the side flowers have bud and semi-open shapes, and **(D)** the central flower and the side flowers have a fully open shape.

**FIGURE 7:**
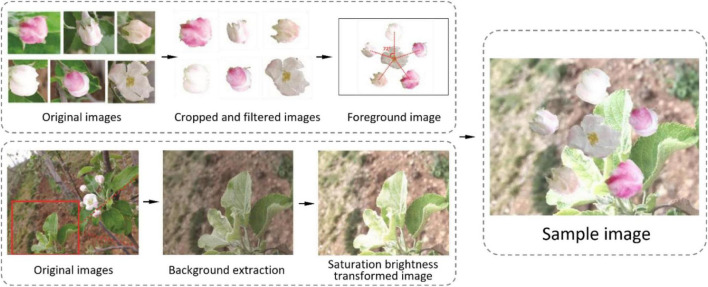
Procedure of image generation in Tian et al. (2020).

Some researchers processed images from angle, brightness, and sharpness to simulate different light conditions (Jia et al., 2020). Some used clockwise rotation, horizontal mirror, color balance processing, and blur processing to augment a data set for apple detection (Tian et al., 2019). Flowers have distinct characteristics from fruit organs. Thus, Tian et al. (2020) proposed a novel image augmentation method as per apple inflorescence (Figure 6). The procedure of image generation is displayed in Figure 7. They clipped 50 pictures of central flowers and 150 pictures of side flowers. Then, they filtered and combined these clipped images to generate foreground pictures. At the same time, 200 pictures were extracted and processed for background pictures. Finally, sample images were produced by coalescing foreground pictures and background pictures. The experiment results proved that this way of augmentation contributed to detection performance.

### Convolutional Neural Network Model Training

#### Training Tools

It is onerous to construct a deep learning model from zero. Many open-source or commercial deep learning tools came into being with the advent of deep learning (Li et al., 2021). In the field of fresh fruit detection, Caffe, TensorFlow, Keras, and PyTorch are popular open-source training tools.

Caffe is the abbreviation of convolution architecture for feature extraction, and is one of the earlier DL frameworks. Caffe defines a network structure in the form of configuration text instead of code. Users can expand new models and learning tasks with its modular components (Jia et al., 2014). TensorFlow is an open-source machine learning library from Google Brain that can be used for a variety of deep learning tasks, including CNN, RNN, and GAN (generative adversarial network) (Abadi et al., 2016). It uses data flow graphs to represent calculations, shared states, and operations (Zhu et al., 2018). Keras is a very friendly and simple DL framework for beginners. Strictly speaking, it is not an open-source framework but a highly modular neural network library based on TensorFlow and Theano. PyTorch is a DL framework launched by Facebook in 2017 and is based on the original Torch framework; it utilizes Python as main development language (Paszke et al., 2019). Furthermore, the open-source code of Caffe2 has merged into PyTorch, which signifies that PyTorch has strong capacity and flexibility. Table 3 describes the detail and differences of the above DL tools. In Table 4, we display the code of the first convolutional layer of Lenet-5 in different languages.

**TABLE 3:** Comparison of Caffe, TensorFlow, Keras, and PyTorch.

Name	Caffe	TensorFlow	Keras	PyTorch
Support language	C++/Python/MATLAB	C++/Python	Python	Python
Support hardware	CPU/GPU	CPU/GPU/Mobile	CPU/GPU/Mobile	CPU/GPU
Support system	Linux/Windows/MacOS	Linux/Windows/MacOS/Android/IOS	Linux/Windows/MacOS/Android/IOS	Linux/Windows/MacOS
Traits	Strong readability and expansibility, stable and superior performance	Comprehensive functionality, good visualization, and active user community	Highly modular, keeping each module short and simple, and ease of extension.	Intuitive design, ease of use, and active user community

**TABLE 4:** Different languages define the code of the first convolution layer of Lenet-5.

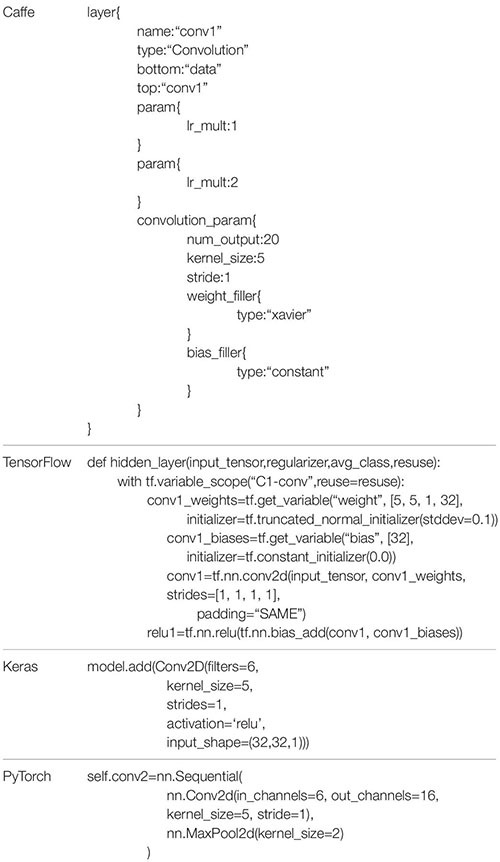

Furthermore, data set annotation, which generates ground truth for supervising networks’ learning object features, is a prerequisite for tasks of object detection and segmentation. Familiar label tools have LabelImg, LabelMe (Russell et al., 2007), Matlab, Yolo_mark, Vatic, CVAT, etc.

#### Parameter Tuning

Parameter initialization is very important. Reasonable initial parameters can help a model improve training speed and avoid local minima. The Kaiming initialization and Glorot initialization methods are generally used (Glorot and Bengio, 2010; He et al., 2015).

In the beginning of the training, all parameters have typically random values and, therefore, far away from the final solution. Using a too-large learning rate may result in numerical instability. We can use warm-up heuristic (He et al., 2019) to gradually increase the learning rate parameter from 0 to the initial learning rate, and then use the conventional learning rate attenuation scheme. With the progress of training, a model will gradually converge to the global optimum. It is necessary to reduce the learning rate to prevent a model from oscillating back and forth near the optimum. Generally, learning rate adjustment strategies such as Step, MultiStep, and exponential and cosine annealing can be used.

Selection of an optimizer plays an important role in DL training and is related to whether the training can converge quickly and achieve high accuracy and recall. Commonly used optimizers include gradient descent, momentum, SGD, SGDM, Adagrad, Rmsprop, Adam, etc.

Convolutional neural network learning needs to establish millions of parameters and a large number of labeled images. If the amount of data is not enough, a model will be over fitted, and the effect is likely to be worse than traditional manual features. If the data set of a new task is significantly different from the original data set and the amount of data is small, one can try transfer learning to complete the new task (Oquab et al., 2014). The weight update of a whole network can be adopted during transfer learning.

### Evaluation Metrics

**FIGURE 8:**
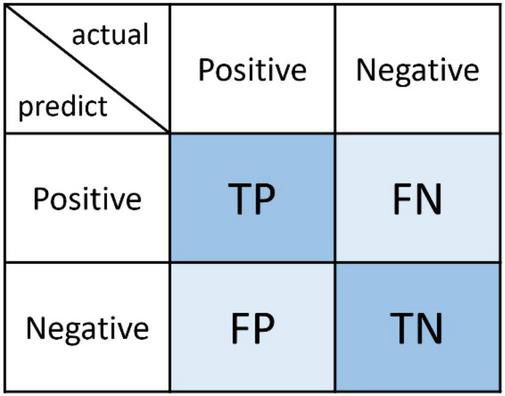
Basic confusion matrix.

The confusion matrix is a basic, intuitive, computational, and simple method for measuring the accuracy of a model. Take the binary classification model as an example, and its confusion matrix is shown in Figure 8. It is mainly composed of four basic indicators: TP (true positive), FN (false negative), FP (false positive), and TN (true negative).

• TP: an outcome where a model correctly predicts a positive class.
• FP: an outcome where a model incorrectly predicts a positive class.
• TN: an outcome where a model correctly predicts a negative class.
• FN: an outcome where a model incorrectly predicts a negative class.

With a confusion matrix, accuracy, precision, recall, and F1-score can be calculated to evaluate a model. Accuracy (Eq. 1) indicates the proportion of correctly classified test instances to the total number of test instances. Precision (Eq. 2) represents the correct proportion of positive samples predicted by a model. Recall (Eq. 3) represents the proportion of all positive samples that are correctly predicted by a model. Generally speaking, precision and recall is a pair of contradictory indicators. As the weighted harmonic average of the two of them, F1-score (Eq. 4) balances the relative importance between precision and recall.


(1) $$A\,c\,c\,u\,r\,a\,c\,y=\frac{T\,P+T\,N}{T\,P+T\,N+F\,P+F\,N}$$

(2) $$P\,r\,e\,c\,i\,s\,i\,o\,n=\frac{T\,P}{T\,P+F\,P}$$

(3) $$R\,e\,c\,a\,l\,l=\frac{T\,P}{T\,P+F\,N}$$

(4) $$F\,1=\frac{2\,T\,P}{2\,T\,P+F\,P+F\,N}$$

In addition to the above basic evaluation metrics, there are also IoU (intersection over union) and mAP (mean average precision) for evaluating the accuracy of a bounding box in an object detection and segmentation model, FPS for detection of speed, and the metrics of the regression model of MAE (mean absolute error), MSE (mean square error), RMSE (root mean square error), and *R*^2^ coefficient of determination, etc. Diversified evaluation indicators can help researchers evaluate and improve algorithms used in many aspects.

ROC curve is often used for evaluating two classifiers. The vertical axis of the ROC diagram is TPrate (Eq. 5) and the horizontal axis is FPrate (Eq. 6). FPrate represents the probability of misclassifying negative cases into positive cases, and TPrate represents the probability that positive cases can be divided into pairs. Each discrete classifier produces an (FPrate, TPrate) pair corresponding to a single point in ROC space. Several points in the ROC space are important to note. The lower left point (0, 0) represents the strategy of never issuing a positive classification; such a classifier commits no false positive errors but also gains no true positives. The opposite strategy of unconditionally issuing positive classifications is represented by the upper right point (1, 1). The point (0, 1) represents perfect classification (Fawcett, 2006).


(5) $$T\,P\,r\,a\,t\,e=\frac{T\,P}{T\,P+F\,N}$$

(6) $$F\,P\,r\,a\,t\,e=\frac{F\,P}{F\,P+T\,N}$$

In addition to ROC curve, MCC (Eq. 7) is also used to measure the performance of binary classification. This indicator considers true positives, true negatives, false positives, and false negatives. It is generally considered to be a relatively balanced indicator. It can be applied even when sample sizes of two categories are very different (Supper et al., 2007). MCC is essentially a correlation coefficient between actual classification and prediction classification, and its value range is [−1, 1]. When it is 1, it means perfect prediction of a subject; when it is 0, it means that the predicted result is worse than the random prediction result; −1 means that the predicted classification is completely inconsistent with the actual classification.


(7) $$M\,\text{CC}=\frac{\text{TP}\times \mathrm{TN}-\mathrm{FP}\times \text{FN}}{\sqrt{\left(\mathrm{TP}+\mathrm{FP}\right)\,\left(\mathrm{TP}+\mathrm{FN}\right)\,\left(\mathrm{TN}+\mathrm{FP}\right)\,\left(\mathrm{TN}+\mathrm{FN}\right)}}$$

## Convolutional Neural Network-Based Fresh Fruit Detection

### Fruit Flower Detection

Fruit flowers are the primary form of fruit organ. Most fruit trees bloom far more than final fruits. However, if there are too many flowers, nutrition supply will be insufficient, which will not only affect the normal development of fruits but will also cause formation of many small fruits and secondary fruits. Yield and economic benefits will be affected. Therefore, flower thinning is necessary to remove some excessive flowers and obtain high-quality fruits (Wouters et al., 2012). After flower thinning, flower detection is implemented and plays a considerable role in fresh fruit production. Flowers of most kinds of fruits are small and dense, resulting in overlap and blockage, which seriously affect the accuracy of detection. Precise estimation based on DL can assist orchardists in assigning labor resources on time to attain a highly effective but low-cost harvest.

The size of flowers of most species of fruits is small, and the flowers are dense, which causes overlap and occlusion quickly. Many researchers detect the flowers in outdoor fields close to make the most of flowers’ traits. Being inspired by the performance of CNNs in computer vision tasks, Dias et al. (2018) incorporated CNN and SVM for apple flower detection. Lin et al. (2020) compared the performance of R-CNN, Fast-R-CNN, and Faster-R-CNN in recognizing strawberry flowers, and Faster-R-CNN ha higher accuracy (86.1%) than R-CNN (63.4%) and Fast-R-CNN (76.7%). Farjon et al. (2020) constructed a system for apple flower detection, density calculation, and flourish peak prediction. The detector in the system was based on Faster-R-CNN. Mask R-CNN with ResNeXt50 is a superior algorithm for recognizing citrus flowers and detecting their quality in an end-to-end model. The average precision of detecting citrus flowers is 36.3, and the error of calculating the number was decreased to 11.9% (Deng et al., 2020). Using U-Net (Ronneberger et al., 2015) as the backbone of Mask-Scoring-R-CNN can also detect flowers with great precision (Tian et al., 2020). At the same time, researchers augmented a data set based on apple flowers’ growth and distribution features to improve the learning capacity of networks. YOlOv4 can detect objects on three different scales. Wu D. et al. (2020) proposed a channel-pruning algorithm based on the YOLOv4 model. The pruned model contains simple structures and has fewer parameters, and it works with sound accuracy and faster speed.

Grape flower counting is often very time-consuming and laborious because the grape flower has particular phenotypic traits that their shapes are the small sphere and growing on the inflorescence densely. Hence, scholars utilized full convolution net (FCN) to detect and identify inflorescences, and then used CHT to recognize the flowers (Rudolph et al., 2019). Palacios et al. (2020) also detected inflorescences and flowers, but both steps used the SegNet architecture with a VGG19 network. In addition, they estimated the actual number of flowers from the number of detected flowers by training a linear regression model. Litchi flowers are also densely clustered and difficult to distinguish in morphology. Thus, a semantic segmentation net that constituted of a backbone net, DeepV3, for feature extraction and a full convolutional net for pixel prediction can detect litchi flower at the pixel level (Xiong et al., 2021).

### Growing Fruit Detection

#### Terrestrial Platform

In addition to fruit flower detection, fruit detection and counting are also important for yield estimation. Fruit growth in fruit trees is different, and fruit thinning needs to be implemented to remove small fruits, residual fruits, diseased fruits, and fruits with incorrect shapes, so that fruits are evenly distributed in trees and branches and can fully receive nutrients. After the fruit thinning and fruit dropping stages, fruits can be detected during fruit ripening to estimate yield (Zhou et al., 2012).

The CNN algorithm has better performance for detecting expanding fruits in a vast scene, which has been proved by comparing it with existing methods (Bargoti and Underwood, 2017). Various species of fruits have different characteristics; therefore, different CNN models are used. Tu et al. (2020) proposed a MS-FRCNN model to estimate passion fruit production. To detect fruits of small and dense olive, researchers tested five different CNN configurations in an intensive olive orchard, and the model with Inception-ResNetV2 showed the best behavior (Aquino et al., 2020). Behera et al. (2021) proposed a Faster-R-CNN model with MIoU, and it achieved an F1 score of 0.9523 and 0.9432 for yield estimation of apple and mango in the ACFR data set. Janowski et al. (2021) employed the YOLOv3 network to predict the yield of an apple orchard. Nevertheless, all algorithms face the problems of occlusion resulting from leaves or branches and fruit overlap. To suppress the disturbance from occlusion, an instance segmentation neural net based on Mask-R-CNN was used to detect apples in two-dimensional space and a multi-view structure from motion (SFM) (Triggs et al., 2002) was used to generate a 3D point cloud according to 2D detection results. Recognizing unripe tomatoes is important for long-term yield prediction, but green fruits are hard to perceive in a green background. Mu et al. (2020) used Faster-R-CNN to detect immature tomatoes in greenhouses and created a tomato location map from detected images. Prediction errors of a whole orchard caused by duplicate statistics attracted the attention of many scholars. It is remarkably effective segmenting individual mango trees with LiDAR Mask and identifying fruits with a Faster-R-CNN-based detector. Koirala et al. (2019a) designed a mango identification system and installed it on a multifunctional agricultural car to realize real-time detection. The algorithm named “MnagoYOLO” in the detction system is modified based on YOLOv2. The car drove on the path between rows of mango trees while the system detected and summed the mangoes on the trees (Koirala et al., 2019a). Some researchers thought of using mobile phones to detect kiwifruits in an orchard in real-time (Zhou et al., 2020). They used a single shot multi-box detector (SSD) with two lightweight backbones, MobileNetV2 and InceptionV3, to develop a device for kiwifruit detection in the wild, the Android app KiwiDetector. Four types of smart phones are used for experiments. Highest detection accuracy can reach 90.8%, and fastest detection speed can reach 103 ms.

**FIGURE 9:**
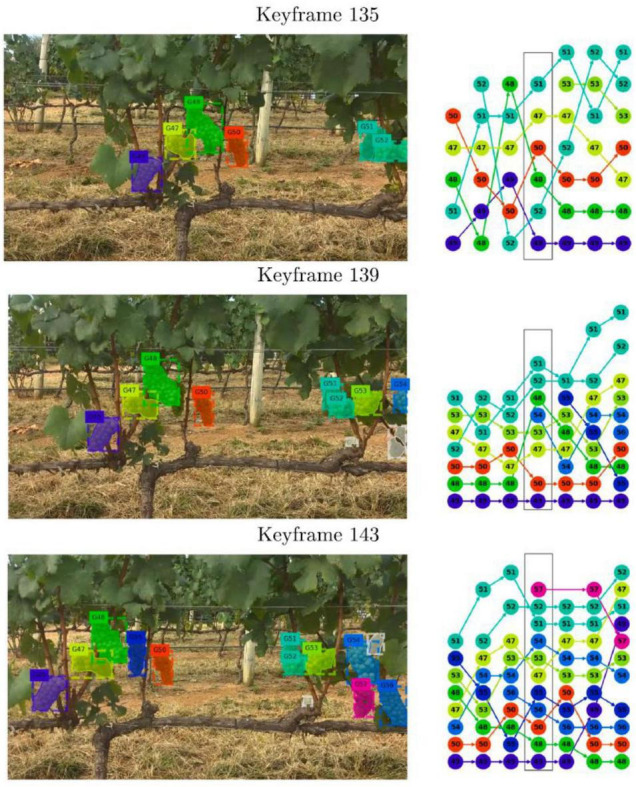
Instance matching and tracking by 3-D assignment. **(Left)** Key frames extracted from a video sequence with a 1,080-p camera. **(Right)** Graph-based tracking. Each column represents instances found by a neural network, and each color represents an individual grape cluster in a video frame.

Deep learning has advantages in yield estimation of clustered fruits. For dense small fruits such as blueberries and small tomatoes, DL has a better detection effect on single fruits and is more convenient for counting fruits. However, using DL to detect small fruits is more vulnerable to the influence of light conditions. To quantify the number of berries per image, a network based on Mask R-CNN for object detection and instance segmentation was proposed by Gonzalez et al. (2019). Grapes are a type of crop presenting a large variability in phenotype. Zabawa et al. (2020) chose to train a CNN to implement semantic segmentation for single grape berry detection, and then used the connected component algorithm to count each berry. SfM (structure-from-motion) can simultaneously solve camera pose and scene geometry estimation to find a three-dimensional structure. Thus, Santos et al. (2020) used Mask-R-CNN to segment grape clusters and generate comprehensive instance masks. Then, the COLMAP SfM software can match and track these masks to reduce duplicate statistics. GPS was employed to establish pair-wise correspondences between captured images and trajectory data (Stein et al., 2016). Figure 9 displays the process of instance matching and tracking. A counting method for cherry tomatoes based on YOLOv4 was proposed by Wei et al. (2021), and it takes the counting problem as detecting and classifying problems that can reduce the effects of occlusion and overlap. Ni et al. (2021) proposed a method for counting blueberries based on the result of individual 3D berry segmentations. In that study, Mask-R-CNN was used for 2D blueberry detection, and the 3D point was used for 3D reconstruction.

**FIGURE 10:**
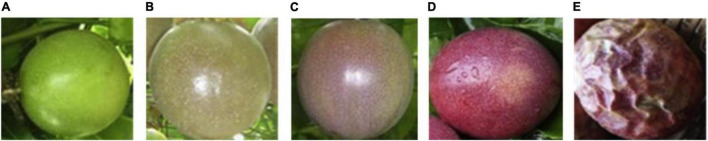
Different maturity levels of passion fruit in Tu et al. (2018). **(A)** Near-young passion fruit. **(B)** Young passion fruit. **(C)** Near-mature passion fruit. **(D)** Mature passion fruit. **(E)** After-mature passion fruit.

Some types of fruits are only edible when ripe. Therefore, maturity monition can provide a timely signal to harvest workers. Tomatoes have the characteristics of clustered growth and batch ripening. Immature tomatoes contain solanine, which is noxious to the human body. Thus, dozens of studies are related to tomato maturity detection. Sun et al. (2018) first used Faster-R-CNN with ResNet 50 to detect critical organs of tomatoes, and the mAP of the model is 0.907. Subsequently, they improved the FPN model to recognize tomato flowers, green tomatoes, and red tomatoes, and the mAP achieved 0.995 (Sun et al., 2020). Coconuts with different maturities can be sold for various purposes. Therefore, Parvathi and Tamil Selvi (2021) used Faster-R-CNN to detect the maturities of coconuts in trees to decrease economic loss. The definition of mature and immature fruits is the primary issue of maturity detection. Some researchers transformed the identification task into a classification task. According to the relationship between storage time and appearance, tomatoes can be classified into five categories: “Breaker,” “Turning,” “Pink,” “Light red,” and “Red.” A CNN can classify the level of tomato maturity (Zhang L. et al., 2018). Tu et al. (2018) collected five maturities category pictures of passion fruit (Figure 10), and then modified the Faster-R-CNN model to recognize the fruit and its ripeness. Tian et al. (2019) divided objective apples into three classes, young, expanding, and ripe, and optimized the YOLOv3 model with DenseNet for detection. The classification method referred in Tian et al. (2019) was used on litchi (Wang H. et al., 2021). However, litchi fruits are different from apples that are small and dense; thus, Wang adjusted the prediction scale and decreased the weight layers of YOLOv3 to enhance the capacity of the model for compact object detection. Khosravi et al. (2021) coded olives according to their mature stages and varieties, divided them into eight categories, and used a deep convolutional network for detection. The overall accuracy of detection can reach 91.9, and the processing speed on the CPU is 12.64 ms per frame.

**FIGURE 11:**
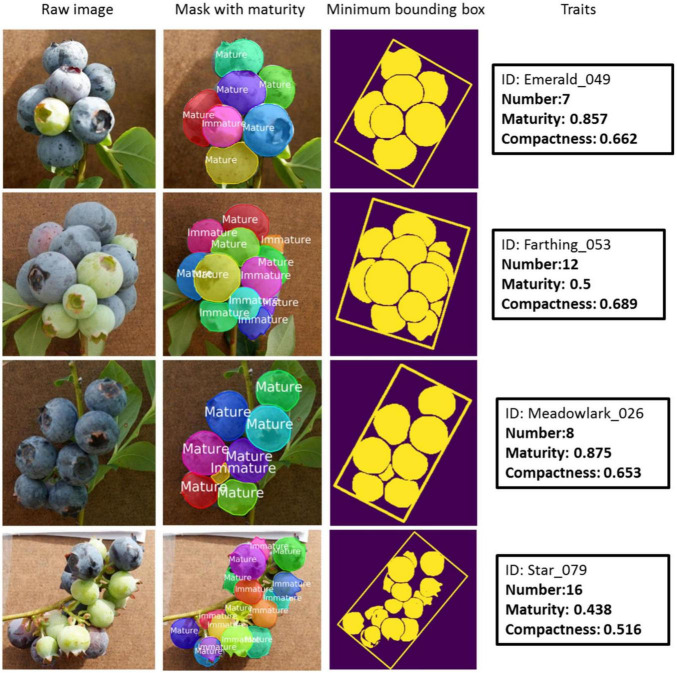
Detection examples in Ni et al. (2020). The black rectangle contains the ID number and three traits (number, maturity, and compactness) of the corresponding sample.

Offering indices of fruit maturity can help workers make harvesting plans and assist harvest robots in making decisions. Some scholars offered indices for describing fruit maturity under the premise of using a CNN to detect fruits. Huang et al. (2020) utilized Mask-R-CNN to identify the location of tomatoes in images and evaluated the HSV value of detected tomatoes. They then constructed Fuzzy inference rules between the maturity and the color feature of the surface of tomatoes, which can predict ripeness and harvesting schedule. Ni et al. (2020) also used Mask-R-CNN to extract blueberry fruit traits and gave two indices to describe fruit maturity (Figure 11). One index is about the maturity of individual berries that can infer whether blueberries are harvestable or not. Another is the maturity ratio (mature berry number/total berry number) of a whole cluster that can indicate the specific harvesting time of this cultivar. For clustered and dense fruits such as blueberries, cherries, and cherry tomatoes, the maturity of whole bunches of fruits can be calculated by detecting the maturity of each fruit using DL. At the same time, the labeling process is time-consuming and laborious. To provide technical support for high quality cherry production, Gai et al., 2021 proposed aYOLO-V4-dense model for detection of the maturity of cherries.

#### Aerial Platform

**FIGURE 12:**
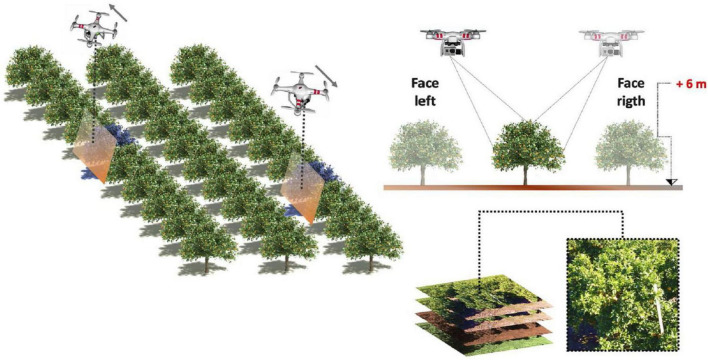
Workflow of field tests (Apolo-Apolo et al., 2020).

Many researchers have begun using UAVs (unmanned aerial vehicles) to obtain images, and UAVs have become common in agricultural remote sensing as intelligent devices progress. Studies have demonstrated that data taken with UAVs are suitable for fruit yield prediction (Wittstruck et al., 2021). Chen et al. (2017) proposed a novel method that uses DL to map from input images to total fruit counts. It utilizes a detector based on an FCN model to extract candidate regions in images, and a counting algorithm based on a second convolutional network that estimates the number of fruits in each region. Finally, a linear regression model maps that fruit count estimate to a final fruit count. A UAV-based visual detection technology for green mangoes in trees was proposed by Xiong J. et al. (2020). In their study, the YOLOv2 model was trained for green mango identification. The mAP of the trained model on the training set was 86.4%, and estimation error rate was 1.1%. Apolo-Apolo et al. (2020) used a UAV to monitor citrus in orchards (shown in Figure 12) and adopted Faster-R-CNN to develop a system that can automatically detect and estimate the size of citrus fruits and estimate the total yield of citrus orchards according to detection results. To solve the problem of inconvenient data capture in mountain orchards, Huang et al. (2022) designed a real-time citrus detection system for yield estimation based on a UAV and the YOLOv5 model. Kalantar et al. (2020) presented a system for detection and yield estimation of melons with a UAV. The system included three main stages: CNN-based melon recognition, geometric feature extraction (Kalantar et al., 2019), and individual melon weight (Dashuta and Klapp, 2018).

After using UAVs to predict fruit yield produced significant results, some scholars began to use UAVs to detect fruit maturity. Chen et al. (2019) used a UAV to capture images of the strawberry crop, and then utilized Faster-R-CNN to detect strawberry flowers and immature and mature strawberries with 84.1% accuracy. Zhou et al. (2021) also divided the growth of strawberries into three stages, “flowers,” “immature fruits,” and “mature fruits,” and utilized the YOLOv3 model to detect images photographed with a UAV. The experimental results show that the model has the best detection effect on the data set taken with the UAV 2 m away from fruits, and the mAP reaches 0.88.

#### Differences Between Two Platforms

In Sections “Terrestrial Platform” and “Aerial Platform,” we have described in detail the existing literature on the use of DL for detecting fruits in the growing period, and the differences can be seen in Table 5.

**TABLE 5:** Summary of related studies on application of CNN-based detection models in growing fruits.

Platform	Purpose	Detected object and label	CNN-based detection model	Following-up works	Remarks	References
Terrestrial platform	Yield estimation	Apple	Mask-R-CNN (2D detection)	SFM photogrammetry is used for generating 3D point cloud and SVM is used for removing false positive	Detection accuracy: 76.2% (2D image detections) and 85.7% (3D detections). Prediction precision: *R*^2^ = 0.8	Gené-Mola et al., 2020
		Apple	YOLOv3	Counting detected fruits for yield estimation	Detection accuracy: 84%	Janowski et al., 2021
		Mango	Faster R-CNN	The GPS/INS, color cameras with strobes, and LiDAR used for fruit locating, tracking, and counting	Prediction accuracy: *R*^2^ = 0.94	Stein et al., 2016
			MangoYOLO	Correction factors are used for estimating yield load	Detection precision: 98.3%. Estimation precision: 4.6–15.2% of packhouse fruit counts	Koirala et al., 2019a
		Tomato	Faster R-CNN	Stitching detected images and compiling a tomato location map of a greenhouse, estimating tomato size as per bounding box size.	Model performance: average precision: 87%, *R*^2^ = 0.87	Mu et al., 2020
		Cheery tomato clusters	YOLOv3	ResNet-50 is used for classifying fruit clusters and counting total fruit number	Prediction precision: RMSE = 6.37, MAPE = 13.9%	Wei et al., 2021
		Passion fruit	Faster R-CNN	Counting detected fruits for yield estimation	Model performance: *P* = 96.2%, *R* = 93.1%, F1 = 0.95	Tu et al., 2020
		Oliver	Inception-ResNetV2	Counting detected fruits for yield estimation	Model performance: F1 = 0.84	Aquino et al., 2020
		Grape clusters	MobileNet-V2	DeepLabV3 segmenting each berry for counting	Berry detection accuracy of 94.0% in the VSP and 85.6% in the SMPH	Zabawa et al., 2020
		Kiwifruit	SSD (with MobileNetV2, quantized MobileNetV2, InceptionV3, and quantized InceptionV3)	Performing on mobiles with Android system and counting detected fruits for yield estimation	True detected rate (TDR) of MobileNetV2, quantized MobileNetV2, InceptionV3, and quantized InceptionV3 are 90.8%, 89.7%, 87.6%, and 72.8%, respectively.	Zhou et al., 2020
		Blueberry	Mask-R-CNN	Using different backbones: ResNet101, ResNet50 and MobileNetV1 to Mask-R-CNN and adding a step to outputs each instance of a blueberry to quantify the total number of blueberries in an image.	The best result was obtained when the ResNet50 backbone was used achieving a mIoU score of 0.595.	Gonzalez et al., 2019
		Blueberry	Mask-R-CNN	3D minimum bounding box calculating fruit cluster compactness after 3D reconstruction and proposing a trait extraction algorithm to segment individual 3D blueberries, count berry number, calculate maturity, and estimate berry size.	The average counting accuracy for the 40 samples is 97.3%. The fruit clusters with a low fruit number generally have a higher accuracy, resulting in almost 100% accuracy.	Ni et al., 2021
		Multi-fruit	Faster-R-CNN with MIoU	Counting detected fruits for yield estimation	Model performance: *R*^2^ of mango, pomegranate, tomato, apple & orange are 0.98, 0.92, 0.96, 0.98, and 0.95	Behera et al., 2021
	Maturity detection	Apple (“Young Apple,” “Expanding apple,” “Ripe apple”)	YOLOv3	Using different data augment methods and data numbers to comparison. Detection under occlusion and overlapping apple conditions and no apple environment.	Model performance: F1 = 0.817. Average detection time: 0.304 s	Tian et al., 2019
		Tomato (“Flower,” “Green tomato,” “Red tomato”)	Faster-R-CNN	Taking comparison between YOLOv2, YOLOv3, original Faster-R-CNN, R-FCN, and proposed model.	Model performance: Mean average precision: 90.7%. Average test time: 0.073 s. Model memory: 115.9 MB	Sun et al., 2018
		Tomato (“Breakers,” “Turning,” “Pink,” “Light red,” “Red”)	Own model	Using own designed CNN for images classification	Classification accuracy: 91.9%	Zhang L. et al., 2018
		Tomato (“Immature,” “Breaker,” “Preharvest,” “Harvest”)	Fuzzing Mask-R-CNN	Locating the stalk points of ripe tomatoes by obtaining the contours of tomatoes from Mask-R-CNN for harvesting.	Model performance: *P* = 96.1%, *R* = 95.9%.	Huang et al., 2020
		Four blueberry cultivars (“Immature” and “Mature”)	Mask-R-CNN	Defining and calculating blueberry maturity and compactness. Assessing the extracted traits and delineating trait differences in four blueberry cultivars.	Model performance: Mean average precision: 78%. *R*^2^ of four cultivars: 0.932, 0.877, 0.859, 0.934.	Ni et al., 2020
		Coconut (“coconut” and “Mature coconut”)	Faster-R-CNN	Comparing the performance of Faster-R-CNN with different backbones, comparing the performance of improved model and other objection detection models.	Model performance: Mean average precision: 89.4%. Detection speed: 3.124 s	Parvathi and Tamil Selvi, 2021
		Passion fruit (“After-mature,” “Mature,” “Near-mature,” “Near-young,” “Young”)	Faster R-CNN	Using DSIFT algorithm and LLC algorithm to extract the features of fruit from R, G, B channels and send the representative features to SVM classifier for maturity indentation.	Detection accuracy: 92.71% and maturity classification accuracy: 91.52%	Tu et al., 2018
		Litchi (“Ripe litchi,” “Expanding litchi,” “Young litchi”)	YOLOv3-Litchi	Comparing the proposed model with YOLOv2, YOLOv3, and Faster-R-CNN.	Model performance: average detection time: 0.029 s, mean average precision: 75.6%, average precision of young litchi, expanding litchi, and expanding litchi is 67.3%, 71.9%, 73.8%.	Wang H. et al., 2021
		Oliver (“ZIG,” “RIG,” “ZVS,” “RVS,” “ZBS,” “RBS,” “ZOR,” “ROR”)	Own model	Evaluating the efficiency of six optimizers: Adagrad, SGD, SGDM, RMSProp, Adam, and Nadam.	Overall accuracy 91.91%, detection speed: 12.64 ms/frame (CPU)	Khosravi et al., 2021
		Strawberries (“Flower,” “Flower-Fruit,” “Green-Fruit,” “Green-White-Fruit,” “White-Red-Fruit,” “Red-Fruit,” and “Rotted-Fruit”)	YOLOv3	Identify the different ripeness of the detected fruit.	The mAP of strawberry maturity classification was 0.89, and the highest classification AP was 0.94 for fully matured fruit.	Yue et al., 2020
		Cherry (“Cherry,” “Cherry_1,” “Cherry_2”)	YOLOv4	DenseNet is used to replace the CSPDarkNet53 in YOLO-V4 and comparing different models in detecting ripe cherries	The mAP increased 0.15 comparing with the YOLO-V4 model and the F1 scores, IOU is 0.947 and 0.856.	Gai et al., 2021
Aerial platform	Yield estimation	Apple, orange	FCN	A second neural network and a linear regression were used to count the number of fruit.	Mean IU of 0.813 on the oranges and 0.838 on the apples, a best l2 error of 13.8 on the oranges, and 10.5 on the apples	Chen et al., 2017
		Green mango	YOLOv2	Counting detected fruits for yield estimation.	The mAP was 86.4%, a precision was 96.1% and a recall rate was 89.0%.	Xiong J. et al., 2020
		Citrus	Faster-R-CNN	Counting detected fruits and estimate the weight for yield estimation.	Mean error is 7.22%.	Apolo-Apolo et al., 2020
		Citrus	YOLOv5	Comparing the proposed model with different models and different occlusion degrees.	Accuracy: 93.32%, speed: 180 ms/frame, FPS: 83 s (In 2080ti), recall: 88.78%	Huang et al., 2022
		Melon	RetinaNet	Estimate the weight of the detected fruit.	Overall average precision score: 0.92 and F1 is more than 0.9	Kalantar et al., 2020
	Maturity detection	Strawberries (“Flower,” “Immature Fruit,” “Mature Fruit”)	YOLOv3	Identify the different ripeness of the detected fruit.	For Flower, Immature Fruit, and Mature Fruit detection from the test data set at 2 m, the APs were 0.83, 0.87, and 0.93, the mAP for the test data set at 2 m was 0.88.	Zhou et al., 2021

From the above discussion, the advantages and disadvantages of terrestrial and aerial platforms for yield estimation and maturity detection are obvious. For orchards located in harsh terrains, it is time-consuming and laborious that researchers use hand-held cameras to obtain data sets, and it is difficult to achieve automatic detection. Researchers only need to remotely control a UAV to easily acquire a large data set with different terrains and shooting distances, which is more convenient than handheld cameras. However, a UAV cannot be too close to the detected subject in the air; otherwise, a collision accident will occur. Therefore, it is noticed that the operation of a UAV needs more skilled technology.

For the yield prediction task, a UAV can capture a wider field of vision, such as fruits at the top of trees. However, when a UAV is used for long-distance shooting, the visibility of fruits is low because fruits at the bottom or inside of a canopy cannot be recognized, and increase in prediction error. When a handheld camera is used, the visibility of fruits is higher because a small part of a blocked fruit can be detected. However, the repetition rate of photographed fruits is high, which is not conducive to yield estimation.

For the maturity detection task, the characteristics of fruits are more conspicuous when a handheld camera is used for close shooting. Fruits photographed with the UAV equipment are too small because of long distance, and the characteristics are relatively fuzzy. In Zhou et al. (2021), researchers used UAV equipment and a handheld camera equipment for data acquisition. They divided the strawberry data captured with the camera into seven different growth stages: flower fruits, green fruits, green-white fruits, white-red fruits, red fruits, and rotten fruits. The strawberry data collected with the UAV were only divided into three labels: flowers, immature fruits, and mature fruits.

### Fruit Picking

The picking period of fruits arrives when fruit organs expand to a certain size. Mature fruits are needed to harvest fruits in time. However, there has been an imbalance between labor force and economic benefits for a long time. In these years, automatic fruit harvest robots have become a hotspot of intelligent agricultural study. Most fruit trees have proper growth heights and structured planting modes that offer convenience to harvest robots. Table 6 summarizes the crops (containing fruits, branches, and trunks) experimented on for automatic harvest and corresponding detection models.

**TABLE 6:** Summary of related studies on application of CNN-based detection models in fruit harvesting.

Crop applied	Basic model	Data augment	Dataset	Transfer learning	Detection rate (%)	Inference speed (s/image)	References
Apple	SSD	√	589 RGB images	√	89.2	–	Peng et al., 2018
	R-CNN	–	270 RGB-D images	√	86.0	–	Zhang J. et al., 2018
	Faster-R-CNN	√	967 three-modalities images (RGB, range-corrected intensity, and depth)	√	94.8	0.074 @548×373 px	Gené-Mola et al., 2019a
	SSD	–	250 RGB-D images	–	92.3	2.00 @3840×1080 px	Onishi et al., 2019
	LedNet (FPN+ASPP)	√	1,100 RGB images	√	85.3	0.028 @320×320 px	Kang and Chen, 2020
	Faster-R-CNN	√	12,800 RGB images	–	87.6	0.241 @1920×1080 px	Gao et al., 2020
	Faster-R-CNN	√	800 RGB-D images	√	87.1	0.124 @1920×1080 px	Fu et al., 2020b
	Faster R-CNN	√	820 RGB images	–	92.5	0.058 @100×100 px	Wan and Goudos, 2020
	Faster-R-CNN	√	675 RGB-D images	√	82.4	0.450 @360×640 px	Zhang et al., 2020
	Mask-R-CNN	–	1,140 RGB images	√	97.3	–	Jia et al., 2020
	Mask-R-CNN	√	24,005 RGB images	√	58.1	–	Dong W. et al., 2021
	Mask-R-CNN	–	19,528 RGB images	–	88.0	0.250 @1280×720 px	Chu et al., 2021
	DenseNet+FPN	√	953 RGB images	√	93.2	0.023 @200×308 px	Xu et al., 2021
Citrus	SSD	√	1,660 RGB images	√	91.1	–	Peng et al., 2018
	Mask-R-CNN	–	300 RGB images	√	85.1	0.045 @1024×768 px	Liu Y. P. et al., 2018
	Mask-R-CNN	√	RGB and RGB-HSV images	√	97.5	0.011 @256×256 px	Ganesh et al., 2019
	Mask-R-CNN	–	5,195 RGB images	√	–	–	Xiong et al., 2019
	Mask-R-CNN	–	750 RGB images	–	98.2	0.700 @1024×768 px	Yang et al., 2019
	Mask R-CNN	–	5,195 RGB images	–	92.2	9.230 @1920×1080 px	Yang et al., 2020
	Faster R-CNN	√	799 RGB images	–	90.7	0.058 @100×100 px	Wan and Goudos, 2020
Kiwifruit	Faster-R-CNN	–	20,160 images	√	92.3	0.274 @2352×1568 px	Fu et al., 2018
	Faster-R-CNN	√	20,160 images	√	87.6	0.347 @2352×1568 px	Song et al., 2019
	Faster-R-CNN	√	21,147 RGB images	√	96.0	1.070 @1920×1080 px	Mu et al., 2019
	Faster-R-CNN	–	1,000 NIR images+1,000 RGB images+1,000 RGB-D images	–	91.7	0.134 @512×424 px	Liu et al., 2019
	YOLOv3	√	20,160 RGB images	√	90.1	0.034 @2352×1568 px	Fu et al., 2021
Strawberry	SSD	√	4,550 RGB images	√	87.7	0.23 @360×640 px	Lamb and Chuah, 2018
	Mask R-CNN	–	2,000 RGB images	√	95.8	0.125 @640×480 px	Yu et al., 2019
	Mask R-CNN	√	–	–	81.0	0.620 @640×480 px	Ge et al., 2019
	Mask R-CNN	–	–	–	–	–	Xiong et al., 2020
	Mask R-CNN	√	3000 RGB images	–	78.3	0.01 @ 1008×756 px	Pérez-Borrero et al., 2020
	FCN		3100 RGB images	–	93.4	0.03 @ 1008×756 px	Pérez-Borrero et al., 2021
Grape	Mask R-CNN	√	1,050 RGB-D images	–	89.5	1.100 @1920× 1080 px	Yin et al., 2021
Litchi	SSD	√	636 RGB images	√	86.7	–	Peng et al., 2018
Mango	Faster R-CNN		822 RGB images	–	88.9	0.058 @100×100 px	Wan and Goudos, 2020
	DenseNet+FPN	√	1694 RGB images	√	93.6	0.023 @500×500 px	Xu et al., 2021
*Rosa roxburghii*	Faster R-CNN	√	8,475 RGB images	–	92.0	0.200 @500×500 px	Yan et al., 2019
Guava	Mask R-CNN	√	304 RGB-D images	√	53.7	0.250 @512×424 px	Lin et al., 2021
Sweet pepper	Faster-R-CNN	√	122 RGB-NIR images	√	83.8	0.393 @–	Sa et al., 2016
	Deep CNN	√	960 RGB images	–	82.9	–	Rehman and Miura, 2021
Cherry tomato	YOLOv3	√	1825 RGB images	–	96.8	0.058 @ 1,292×964 px	Chen et al., 2021

#### Fruit Recognition on Fields

The recognition and detection of fruits in an orchard environment provide robots with vital contextual information for maneuvering. However, branches, foliage, and illumination conditions affect the fruit detection with robots. Feature augmentation is a simple way to enhance the learning capacity of DL models. Mu et al. (2019) collected images with four types of occlusions in four illumination conditions as training data. Some researchers divided target apples into four classes depending on their obscured circumstances: leaf-occluded, branch/wire-occluded, non-occluded, and occluded fruits (Gao et al., 2020). Different varieties of the same fruit will have subtle differences in appearance. Using Mask-R-CNN to segment fruit images can distinguish fruits from occluded ones well. Chu et al. (2021) used an integrated data set with two varieties of apple to train Mask-R-CNN for suppression. Jia et al. (2020) optimized the Mask-R-CNN model in the backbone net, ROI layer, and FCN layer for apple harvesting robots. In a research study on strawberry harvest, the researchers reduced the magnitude of backbone and mask network and used a process of filtering and grouping of candidate regions to replace the object classifier and the bounding box regressor Mask-R-CNN. The new architecture can process original high-resolution images at 10 frames per second (Pérez-Borrero et al., 2020). Then, Pérez-Borrero et al. (2021) proposed a new strawberry instance segmentation model based on FCN whose FPS rate was six times higher than those obtained in reference methodologies based on Mask R-CNN.

**FIGURE 13:**
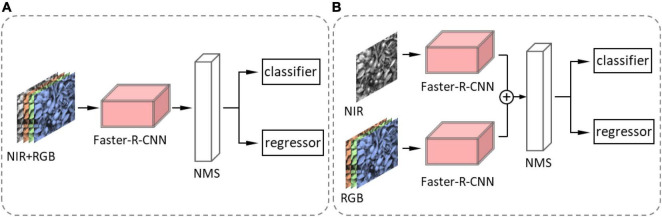
Diagram of fusion methods in Sa et al. (2016). **(A)** Early fusion: first, channels of the detected image are augmented from three to four channels. Second, the augmented image is detected by Faster-R-CNN. Third, NMS (non-maximum suppression) removes duplicate predictions. Finally, the classifier and regressor calculate the category and coordinate of the bounding box. **(B)** Late fusion: first, the RGB image and the NIR image are detected by Faster-R-CNN. Second, the detected outputs from two Faster R-CNN networks are fused. Third, NMS (non-maximum suppression) removes duplicate predictions. Finally, the classifier and regressor calculate the category and coordinate of the bounding box.

**FIGURE 14:**
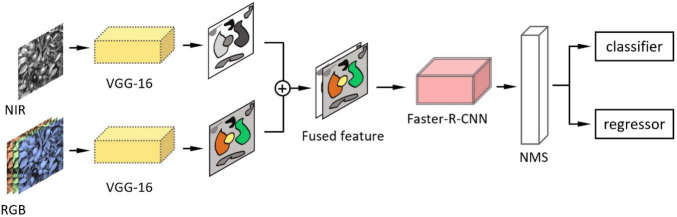
Feature-fusion model in Liu et al. (2019). First, it inputs the RGB and NIR images separately into two VGG16 networks and then combined them on the feature map; then, the feature map is detected by Faster-R-CNN.

As we have discussed in Section “Dataset Acquisition,” a depth image contains more information. Ganesh et al. (2019) assessed the performance of Mask-R-CNN by applying three forms of color space input, RGB images, HSV images, and RGB + HSV images. The result showed that adding HSV information to RGB images can decrease false positive rate. Sa et al. (2016) explored two methods for imagery modality fusion based on Faster-R-CNN. One is early fusion (Figure 13A) by augmenting channels of input images from three (red, green and blue) to four (red, green, blue, and NIR) channels. Another is later fusion (Figure 13B) that fuses pieces of classified information of an RGB-trained model and an NIR-trained model. NIR (near infrared) here refers to images taken by near-infrared imaging technology. There are also two fusion methods for detecting kiwifruits based on Faster-R-CNN (Liu et al., 2019). One is similar to the early fusion (Sa et al., 2016), and the other fuses the feature maps from two modes displayed in Figure 14. The background objects of RGB-D images captured with a Kinect V2 camera can be filtered by distance threshold and foreground-RGB images, and Faster-R-CNN with VGG achieved a high average precision of 0.893 for the foreground-RGB-images (Fu et al., 2020b). Gené-Mola et al. (2019b) added an imaging modality, the range-corrected IR intensity proportional to reflectance, based on RGB-D images. It makes an input image become five channels, and the F1-score of the detection model improves 4.46% more than simple RGB images.

In most studies, researchers spent energy optimizing algorithms. Peng et al. (2018) used SDD and replaced the original VGG-16 with ResNet-101 to detect apple, citrus, and lichi. Besides, decreasing layers of the backbone of SSD can achieve accurate and precise detection in a low-power hardware (Lamb and Chuah, 2018). Kang and Chen (2020) designed a CNN model named “LedNet,” which is mainly improved by a lightweight backbone, FPN, and ASSP, for fruit detection in an apple orchard. Integration of DenseNet and FPN can obtain small fruits’ features more correctly (Xu et al., 2021). Fu et al. (2018) first used a DL model for kiwifruit detection in 2018, and they developed a kiwifruit detection system based on Faster-R-CNN with ZFNet for filed images. Three years later, they proposed a DY3TNet model based on the addition of convolutional layers to YOLOv3-Tiny for kiwifruit recognition in a wild environment (Fu et al., 2021). Some scholars are also dedicated to kiwifruit detection but used Faster-R-CNN with VGG-16; however, the precision and speed of detection are lower than the results of Fu et al. (2018). Modification of the pooling layer can also improve detection accuracy. Yan et al. (2019) changed the Faster-R-CNN model by replacing the ROI pooling layer with the ROI align layer. Wan and Goudos (2020) modified the pooling layers and convolution layers of the existing Faster-R-CNN. In the two experiments (Yan et al., 2019; Wan and Goudos, 2020), detection speed and accuracy accomplished prominent improvements. As we know, most fruits are elliptical in a 2D space. Thus, specialists presented an ellipse regression model based on Mask-R-CNN for detecting elliptical objects and inferring occluded elliptical objects (Dong W. et al., 2021). The original YOLOv3 has low precision in detecting cherry tomatoes, and DPNs (dual-path networks) can extract richer features of recognition targets. Therefore, researchers improved the YOLOv3 model based on DPNs for identification of cherry tomatoes.

#### Obstacle Avoidance

Robots should also learn to avoid foliage and branches except when identifying fruits. For sure, researchers thought of making robots recognize obstructions while detecting fruits, so robots can react differently according to different objects. Using the R-CNN model to detect and locate branches of apple trees in natural environments can establish a branch of skeletons, so that the arms of robots can avoid branches while grabbing apples (Zhang J. et al., 2018). For citrus harvest, Yang et al. (2019) utilized the Mask-R-CNN model to recognize and reconstruct branches of citrus trees. Later, the researchers designed a recognition model based on their previous studies for citrus harvest robots to detect fruits and branches simultaneously (Yang et al., 2020). Lin et al. (2021) used a tiny Mask-R-CNN model to identify fruits and branches of guava trees and reconstructed the fruits and branches for robotic harvest.

**FIGURE 15:**
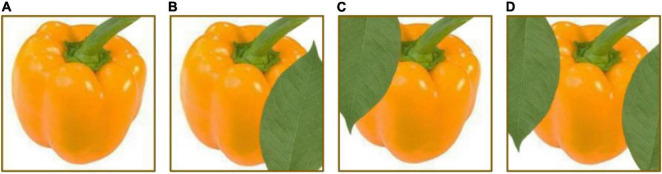
Possible types of fruit in one scene formulated by Rehman and Miura (2021). **(A)** Center, **(B)** left, **(C)** right, and **(D)** occluded.

There are some other means for occlusion avoidance except when detecting obstructions. Rehman and Miura (2021) presented a viewpoint plan for fruit harvest. They demonstrated the possible types of a fruit in one scene with the labels “center,” “left,” “right,” “occluded,” which are depicted in Figure 15. The arm of a robot is qualified to determine the harvesting path as per detected labels. What is more, objective fruits could be classified into normal, branch occlusion leaf occlusion, slight occlusion overlapping, or main branch (Liu Y. P. et al., 2018). Also, a new strawberry-harvesting robot with a more sophisticated active obstacle separation strategy has been developed, and the strawberry location detector in the system is based on Mask-R-CNN (Xiong et al., 2020).

#### Picking Point Detection

The feasibility of automatic harvesting has been confirmed broadly. A further important issue is locating harvesting points precisely that can guarantee that the robot’s grasp of fruits is accurate and uninjurious. Mask-R-CNN not only can detect an object accurately but can also generate corresponding masks of an object region at the pixel level, which can assist in locating picking points. Longye et al. (2019) segmented and reconstructed the overlapping citrus using the Mask-R-CNN model and performing concave region simplification and distance analysis. Strawberry detection can also employ the Mask-R-CNN model. Then, picking points are determined by analyzing the shape and edge of objective masks (Yu et al., 2018). Ge et al. (2019) also utilized the Mask-R-CNN model to detect strawberries based on RGB-D images that have depth information of images; they performed coordinate transformation and density-based point clustering, and proposed a location approximation method to help robots locate strawberry fruits. Yin et al. (2021) proposed segmenting the contours of grapes from RGB images with Mask-R-CNN and then reconstructing a grape model by fitting a cylinder model based on point cloud data extracted from segmented images. By recognizing and calculating the outline of a bunch of grapes, the arm of robot can grab stalks at the top of a bunch of grapes. Shake-and-catch harvesting first appeared in 2010 (He L. et al., 2017). Some researchers used the Faster-R-CNN model to establish a relationship between fruit location and branch location (Zhang et al., 2020). Connections can help a robot to determine shake points.

**FIGURE 16:**
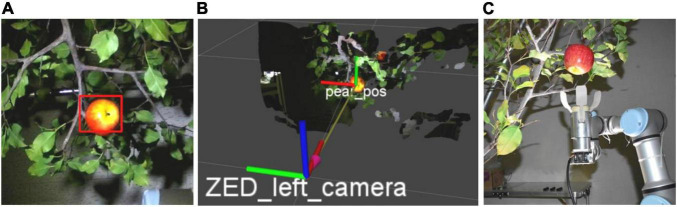
Automatic apple harvesting mode in Onishi et al. (2019). **(A)** Detection of a two-dimensional position, **(B)** detection of a three-dimensional position, **(C)** approaching the target apple.

Generally, researchers detect fruits on the side of trees, but Onishi et al. (2019) proposed a novel method for inspecting apples from below. The SSD model is used to detect the 2-D position of the apple shown in Figure 16A. The stereo camera ZED provides the 3-D position of the center of the bounding box, which is like in Figure 16B, and the position can be a picking point. Then, the robot can move below the target apple to grasp the fruit according to the predicted position like in Figure 16C.

### Fruit Grading

After a fruit is picked, it will gradually flow to the market and produce economic benefits. Recently, customers have higher requirements for fruit quality as consumption levels increase. Hence, it is necessary to evaluate the quality of fruits before delivering them to consumers because of external and internal vulnerabilities. Those with better fructifications can be consumed, and those with worse can be processed to make fruit foods. Graded-based vendition by detecting internal diseases, sugar content, surface damages, maturity, size, etc. can promise both seller and purchaser benefits. In this section, we will introduce the research on CNN-based fresh fruit grading from grading as per external traits, grading as per internal traits, and fruit cultivar classification.

#### External Trait-Based Grading

External phenotypic characteristics of fruits directly show their qualities, which affect the sale price and consumer enthusiasm. Thus, external quality detection plays a significant role in fruit grading. Many experiments testified that CNNs have noteworthy superiority in fruit quality grading (Wang et al., 2018; Jahanbakhshi et al., 2020; Patil et al., 2021). In the research of Wang et al. (2018), a modified AlexNet model was used to extract the feature of defects on litchi surface and classify litchi defect images. The classification precision of the AlexNet-based full convolutional network is higher than that of linear SVM and Naive Bayes Classifier. Jahanbakhshi et al. (2020) compared sour lemon detection performance based on a CNN model with other image categorization methods and demonstrated the superiority of the CNN-based model in fruit grading. Patil et al. (2021) also concluded that CNNs have a faster speed of operation in dragon fruit grading and sorting by comparing the performance of ANN, s, and CNN models.

**FIGURE 17:**
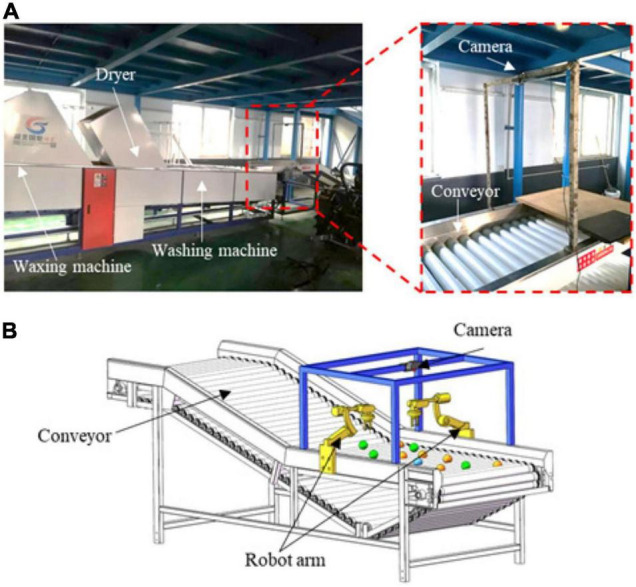
Platform setup and computer vision system (Chen et al., 2021). **(A)** The citrus processing line was assembled in the laboratory, with a webcam mounted above the conveyor. **(B)** The diagram shows an automated citrus sorting system using a camera and robot arms, and the robot arms will be implemented in future studies.

Apple is the most salable and lucrative fruit globally. Some researchers developed apple defect detection systems for apple grading. Fan et al. (2020) designed a 4-lane fruit sorting system to detect and sort defective apples, and a CNN model for a defective apple sorting system, in which a global average pooling layer was applied to replace a fully connected layer. Wu, Zhu, and Ren performed laser-induced light backscattering imaging to capture apple defect images and designed a simple CNN model to classify scabs on apple surface (Wu A. et al., 2020). Aside from scabs on apple surface, the CNN model can classify images of apples with bruises, cracks, and cuts (Nur Alam et al., 2020). Researchers also conducted related studies on other fruits. Azizah et al. (2017) used a CNN model to implement mangosteen surface defect detection. Zeng et al. (2019) constructed an ensemble-convolution neural net (E-CNN) model based on the “Bagging” learning method for detection of defects in jujube fruits. Cherries are prone to abnormal shapes during growth, so some researchers used a modified AlexNet model to classify cherries according to growth shapes (Momeny et al., 2020). Wu S. et al. (2020) combined and investigated several deep learning methods for detecting visible mango defects and found that VGG-16 has a dominant position by combining and investigating several DL methods. De Luna et al. (2019) also demonstrated that the VGG-16 model has better performance in tomato defect inspection. Some researchers used a modified ResNet-50 model to extract the features of tomato surface defects and classify images of tomato defects (Da Costa et al., 2020). Chen et al. (2021) established an online citrus sorting system, shown in Figure 17, and a detector named Mobile-citrus based on Mobile-V2 to identify surface defects in citrus. Then, the arms of robots arms pick out the defective ones with the linear Kalman filter model used in predicting the future path of the fruits.

The external appearance of a fruit sometimes also represents its freshness. A multi-class classifier based on VGG-16 and Inception-V3 was built by Ashraf et al. (2019) for detecting fresh and rotten fruits. Researchers also practiced the advantages of CNNs in classifying the freshness of apples, bananas, and oranges (Ananthanarayana et al., 2020).

#### Internal Trait-Based Grading

Commonly used RGB images cannot acquire internal traits of fruits, for instance, diseases, sugar content, moisture, etc. Consequently, many researchers combined CNN-based DL models with spectrum techniques and made remarkable progress in internal quality-based grading. The sweetness, crispiness, and moisture of apples can be detected using hyperspectral images and 3D-CNN (Wang et al., 2020). Researchers have also proposed a multi-task model based on 3D-CNN for predicting the sugar content and hardness of yellow peaches simultaneously (Xu et al., 2020). Jie et al. (2021) proposed a non-destructive determination method based on the YOLOv3 algorithm, and hyperspectral imaging technology contraposes citrus granulation.

## Challenges and Future Perspective

As per the above statements, the appearance of CNN models is already invigorating the automatic production of fresh fruits. However, people remain having quite a lot of challenges to face, because the whole automation of the fruit industry is merely in the period of development.

### Environmental Issues

The problem of fruits being occluded is a difficulty in fruit detection. Most occlusions are caused by foliage, branches, trunks, and fruit overlapping in complex fruit-growing environments. Moreover, varying illumination conditions are also one of the instability factors in fruit detection. For instance, green fruits, such as green citrus, green litchi, avocado, and guava, conceal in a green background, which results in more faulty detections of machine visions. Thus, algorithms with high detection accuracy and speed are the objective of researchers.

In addition to algorithm improvement, human intervention can also assist in solving environmental issues. It is a feasible method to increase the visibility of fruits by trimming the crown of fruit trees and standardizing planting according to the principles of horticultural operations. For example, a trellised fruiting wall is suitable for robotic operations during pruning and harvesting (Majeed et al., 2020). Artificially improving the lighting of an environment can also reduce uncertainty in the process of detection. When light is strong, cameras are prone to overexposure. In response to this problem, some researchers have adopted a shading platform to reduce the impact of sun exposure (Gongal et al., 2016; Nguyen et al., 2016; Silwal et al., 2016). To increase the utilization rate of machines, people will have to let robots work at night. However, there is insufficient lighting during night operations, and external light sources are needed to improve the lighting of an environment (Koirala et al., 2019a). Most of the current shading devices and light supply devices are relatively bulky, so it is of commercial value to design a shading or a lighting system that is simpler and more portable.

### Exploration of New Areas

In the process of fresh fruit production from blooming to marketing, and pollination, pesticide application, harvesting, sorting, and grading all need a large pool of workers. The preceding discussion suggests that most applications of CNNs in fresh fruit production are in the algorithm development stage. Autonomous operation of robots is mostly used for fruit harvesting and grading. There are fewer exploitations of automatic pollination robots for the problem of greenhouse plants’ insufficient pollination. In current studies, Chunjiang Zhao utilized the improved YOLOv3 network to identify tomato flowers in greenhouses and embedded the system in automatic pollination robots. Phenology distribution monitoring can govern the timing and dosage of chemistry thinning, which determines the quality of fruits. Fruit flower phenology involves a period from the emergence of fruit buds to petal withering means that monitoring of flower phenology is not only estimating flower number. Studies on using computer vision to detect fruit flower phenology are rare, and CNN-based methods are even less. According to our search, Wang X. et al. (2021) designed a phenology detection model based on a CNN named DeepPhenology to estimate apple flower phenology distribution. Currently, more researchers are utilizing CNN to detect fruit flowers and achieve the purpose of yield estimation. Perhaps the application of CNN in fruit flowers phenology estimation is a new area worth exploring.

Food safety is an issue that concerns people, because accumulation of pesticides in the human body risks causing cancers. However, pesticide residues on fruit surfaces are inescapable, because orchardists will perform pesticide delivery to guarantee fruit’s healthy growth. CNNs can be used to identify pesticide residues, but the CNN used in most studies (Yu et al., 2021; Zhu et al., 2021) is a one-dimensional CNN, and input data are pre-processing data extracted with a spectrometer. The process of detection is complicated and cumbersome. Rarely have researchers used the 2D CNN model to detect pesticide residues in harvested fruits (Jiang et al., 2019). Although pesticide residues belong to the external characteristics of fruits, its vision detection still needs hyperspectral images, because RGB images cannot capture pesticide residues. The current detection methods have complex processes out of proportion to the economic benefits generated by pesticide residue detection. Thus, the feasibility of using CNNs to detect pesticide residues in fruits should be studied further. When grading and sorting clustered fruits such as grapes, litchis, and longan, a manipulator grabs the stalk on the top of a fruit to minimize damage to the fruit. However, fruits on the sorting table are arranged disorderly, and stalks are not arranged neatly on a horizontal plane. Therefore, it is necessary to use CNNs to determine the robot’s sequence of grabbing of clustered fruits (Zhang and Gao, 2020).

There is no doubt that CNNs have a developing potential in fresh fruit production. In future studies, it is promising to enhance the application areas of CNNs in fresh fruit detection. It could be a good direction that infuses CNNs into whole fruit production.

### Execution of Multiple Tasks

Fruit surfaces are easily damaged, so the general method is utilizing a mechanical arm to grab fruits to reduce mechanical injuries. Most existing CNN-based picking robots are based on one fruit kind, However, the time of fruit harvest is not continuous, therefore, robots are, most, of the time idle. That generates averse economic effectiveness, because robots have high manufacturing expenses but low use ratio. According to the advantages of CNNs, they can directly extract features from input images; therefore, scholars can develop algorithms that can detect and locate a variety of fruits (Saedi and Khosravi, 2020). The mode of multitask operations can improve the use ratio of harvest robots that ensures fruit harvest robots’ commercial value.

In CNN-based fruit quality grading, detection methods based on RGB images can only identify external defects, and detection methods based on hyperspectral and infrared images are focused more on internal trait detection. Results of a single detection technique are biased. Simultaneous detection of multiple quality parameters and comprehensive evaluation are a good improving trend. In addition, detection algorithms and hardware should be optimized with increasing detection difficulty.

## Conclusion

The perishability and fragility of fruits make fruits use more labor force for careful care during the production process, which is also the reason why most fruits are expensive. At present, many researchers are bringing artificial intelligence into the field of fruit production and are carrying out a series of research studies on the use of machine vision to identify fruits. In this article, the principle of CNNs and implementation of CNN-based detection methods is elaborated, enabling researchers to better understand CNNs and their applications in fruit detection. This review emphasizes the application of CNNs in fresh fruit production, including detection of fruit flowers, detection of fruits in the expansion period, detection of fruits in the harvest period, and detection of fruits before entering the market. We have performed a lot of investigations and analyses of literature in this area and presented in detail the convolution models, improvement points, training methods, detected objects, and final detection results in these studies. Through our investigation of experiments, we found that CNNs do have exceptional performance in the detection of fruits. However, this does not mean that fruit detection should evolve toward a single direction of detection based on CNNs. Through our comprehension and comparison of current research, we summarized the challenges that researchers encountered when using CNNs for fruit recognition and discussed future development trends.

## Author Contributions

CW, JX, and ZZ designed the survey. SL, BZ, LL, GL, YW, and PH collected and analyzed the data, and wrote the manuscript. JX and ZZ revised the manuscript. All authors contributed to the article and approved the submitted version.

## Conflict of Interest

The authors declare that the research was conducted in the absence of any commercial or financial relationships that could be construed as a potential conflict of interest.

## Publisher’s Note

All claims expressed in this article are solely those of the authors and do not necessarily represent those of their affiliated organizations, or those of the publisher, the editors and the reviewers. Any product that may be evaluated in this article, or claim that may be made by its manufacturer, is not guaranteed or endorsed by the publisher.

## Abbreviations

CNN: convolutional neural network
DBN: deep belief network
RNN: recurrent neural network
VGG: visual geometry group
DTI: decision tree induction
SVM: support vector machine
2D: two-dimensional
ms-MLP: multiscale-multilayered perceptron
HoG: histogram of oriented gradient
ML: machine learning
GLCM: gray-level co-occurrence matrix
CIELab: Commission Internationale de l’Eclairage Laboratory
CHT: circular Hough transform
SLIC: simple linear iterative clustering
YOLO: you only look once
SSD: single shot multibox detector
mAP: mean average precision
STN: Special Transform Network
CCD: charge coupled device
SMOTE: synthetic minority oversampling technique
DC-GAN: deep convolutional generative adversarial network
CycleGAN: cycle generative adversarial network
CVAE-GAN: conditional autoencoder generative adversarial network
GAN: generative adversarial network
CPU: central processing unit
GPU: graphics processing unit
TP: true positive
FN: false negative
FP: false positive
TN: true negative
MAE: mean absolute error
MSE: mean square error
RMSE: root mean square error
FCN: full convolutional net
AV: unmanned aerial vehicle
MS-FRCNN: multiple scale Faster R-CNN
MIoU: mean intersection over union
SFM: structure from motion
ROI: region of interest
E-CNN: ensemble-convolutional neural net
NIR: near infrared.
